# Requirements of Teeth Extraction during the Period of Youth

**Published:** 1887-02

**Authors:** J. Hardman

**Affiliations:** Muscatine, Iowa.


ARTICLE III.

### REQUIREMENTS OF TEETH EXTRACTION DURING THE PERIOD OF YOUTH.

BY J. HARDMAN, D. D. S., OF MUSCATINE, IOWA.

The life period of the human is divided into infancy, youth, adolescence and old age. Each of these periods have attending conditions more or less specially marked as individual characteristics belonging to it.

While viewing this question from a general stand-point, the anatomical and physiological structures and functions are found to be in strict harmony with each epoch. The infant has the organism of the growing child; the youth of structural change from infancy to adolescence—adolescence manifesting the full acme of complete manhood; while old age is attended with its marks of decline towards a final close and dissolution of all that went to make up the individual's organism during a lifetime.

The relative diversity in each of these periods, in a general way, is not more marked than they are in a special way. The oral conditions, odontologically considered, are as manifest in their variations throughout life as are the general parts of the being; and hence, as the general treatment is intelligently varied and adapted to suit and correspond to the attending age and conditions, it must follow, ergo, that oral or dental treatment must also agree in kind and variation suitable to each period of the patient's life or stage of development.

While the question as to how extraction of teeth should be conducted in each of these other divisions, as well as this one of youth, would be of much interest to dentinal readers, it is obvious, however, that time or the needs would not at present justify such consideration.

I perceive that the most obvious defects in treatment by needful extraction is more generally prevailing in the profession at this day in patients belonging to the class properly known as youths than any others. I have, therefore, taken this period of life upon which to advance some suggestions. I am aware that some very deserving operators wholly pronounce against extraction as being proper, or required, under *any* circumstances; but, as such advocates are obviously in error, and are yearly growing less in numbers, we conclude there is no need in taking up time in attempting a refutation of their position.

We concede that correct practice will seldom prompt the intelligent operator to remove a tooth or teeth *simply* on account of odentalgia, but because *some other attending condition or conditions demand it*; and some of these attending conditions during youth is what we propose now to consider.

In starting out we must not overlook the fact that everything in such a mouth is yet incomplete and in a state of formative development. The maxillæ are yet expanding; the teeth not fully emerged, and are in a state of incomplete development; the jaw contains more teeth in a state of growth than at any other stage of life; greater sensibility of the entire system prevails, and the patient less qualified to properly care for the teeth, besides many other conditions peculiar to this age that might be mentioned, as sedentary school life, age of puberty, unusual demand for tooth material, etc.

The intelligent practitioner should highly estimate the value that judicious extraction has in this period in preventing and correcting irregularity. While he justly values the worth, and often effectual influence of appliances and means towards expanding and modifying the dimensions of the jaw during this period, and even during adolescence, yet in many cases judicious extraction has greatly the preference in effectiveness, in time, in ease of accomplishment and in expense. It has been fairly accepted as an axiom that the patient may have inherited the small maxillary of one parent and the large size of teeth of the other, and *vice versa*. Hence the deformities of frequent cases of irregularity, of extreme pouching and of over-crowding, are resting upon a natural basis. Any effort to correct any of these evils by appliances, while still retaining all the teeth, is liable to prove a certain failure.

1st. The endurance of the patient to wear such appliances sufficiently constant and protracted, is seldom found.

2d. When accomplished, the constant tendency of nature to reassert its primary principle is very certain to reestablish the same prior conditions—not typical of a complete morphology of a race, but such as was transmitted from parents to the child.

3d. Where irregularities in jaws thus small and of over-sized teeth are attempted to be overcome by a mechanical expanding force, the effort amounts generally only in protruding outwardly the points of the teeth, giving a malposition of the true perpendicular axis of the teeth, causing the force of mastication to come *not* perpendicularly upon the end of the tooth, but obliquely, pushing outwardly, thus greatly lessening the strength and power of the teeth in service, also inducing a disgusting outward projection of the teeth, with facial deformity and early loss of gingival upport.

A judicious thinning out by the removal of one or more teeth, at an opportune time, will generally effectually accomplish the greater amount of good, *immediate and remote*, than can be done without extraction. Of course other means must not be eschewed, as they furnish in many cases important and very efficient aids.

It is not infrequent that we meet one or the other jaw greatly protruding, caused alone by this great disproportion of maxilla and teeth, more frequently the upper. Here the removal of a tooth or two, upon each side of the protruding jaw, will often accomplish wonderful corrective change, both as to symmetry of outline and of usefulness. Again, where this hyper-dental condition is attended with great tendency to caries, a thinning out is imperative, especially if at the same time other demands call for extraction as a remedy and the teeth are crowded in the jaw.

In extracting for any of the foregoing causes, I should, other things being equal, prefer the first bicuspid, next the second bicuspid or first molar—always making an exposed, diseased or dead pulp, or a suppurating tooth, a leading cause to mark the doom of the tooth.

Next we wish to call special attention to the frequent necessity to remove the first molar some time (early) during the period extending from the time this tooth is emerged and the emerging of the third molar, a period well marking the limits of this age of youth. Whenever the pulp of this first molar is exposed, or if it is dead at this age, and especially if the second and third molars are not missing, *treat by extraction*. To settle this our way, we would say, extensive anticipated physiological changes in adjoining parts, as well as pathological conditions of the tooth itself, must exert a large influence in treatment.

The immature state of the tooth, the absolute need of pulp influence, the changes of position in consequence of the coming second and third molars crowding for place—these go far to disqualify this tooth for usefulness and susceptibility of treatment *after* nerve exposure. We think we are safe to say, that where in this tooth a nerve is capped or devitalized, or where the root membranes are suppurating, and this tooth filled any time prior to the emerging of the dens-sapientia, the sequel will prove a failure. Not one in fifty will remain in a useful state at the age of twenty-five. But, the rule will be deterioration; that amounts to disintegration, passing generally into alveolar abscess, and the finale, quite certain, will be the removal of the tooth. It is hardly necessary to say that the loss of nerve influence in cutting off the further supply of mineral and other tooth elements so much needed in this immature tooth, cannot be expected to be supplied through the peridentium. It absolutely requires *nerve* (or pulp) influence to carry on the deposit of tooth element; that where this is cut off, there is an end to further development of this kind. The tax upon vital energy, while the struggle for position and development of the second and third molars will be too great, and pathological action is established, that generally ends in the suffering patient yielding to removal. Where this tooth is removed some time before the emerging of the third molar the second, and finally the third, will gracefully and certainly shift forward and close up the space, securing an excellent tier of grinders and the concentration and conservation of increased vital energy to the third molar, making that a well matured and permanent organ; while upon the other hand, an attempt to save a first molar already in this condition will surely entail unremunerative expense and prolongation of suffering. Despair comes, though sometimes late, but only too sure; the tooth is extracted *after the emerging of the third molar and a permanent gap is the sure result, with the third molar deficiently developed, badly crowded back and out of usefnl range, of but little service and short life.*

The statement by some, that the early extraction of this tooth is followed by a forward dipping position of the second molar, and also of deformity by favoring malformation of the maxillary bones, etc., we can but regard as merely fanciful. The former is much oftener the result of *too late* extraction when it does occur, and the latter is more generally prevented by the loss of this tooth in season, producing improved physiognomic contour rather than damage, as indeed the attempt to save the one is likely to sacrifice the two. The plan and recommendation of removing the entire first four molars, where one is past salvation, in order, as it is claimed, to secure and maintain perfect artic ulating antagonism, is, in my view, bad philosophy and reprehensible practice. By close observation in hundreds of cases where but one of a jaw was removed, we have found nature come to the rescue, and with such complete restitution that we can but regard the wholesale extraction of sound teeth in this case,and alone for this purpose, as erroneous and ill practice.

My observations force me to the conclusion that capping the nerve, under *any* approved plan, will prove a failure in this tooth any time before the third molar is developed ; and if the nerve is devitalized, no mode of root or crown-filling, along with the best therapeutic treatment, will carry the tooth even to adult age and then be worth one-half what it cost in suffering and disappointment; and if persevering endurance has it in possesion at this adult age, ninety-nine chances to one it is more or less a nuisance and will not be tolerated but a limited period, and then its removal be followed by a pernament gap, with very certainly an imperfect third molar of little service to the possessor.

We have thus confined our consideration of the needs of extraction of teeth, during youthful age, to the prevention and correction of irregularities, and to the good influence correct and timely extraction may have in the management of the molars. This, of course, cannot cover all the cases that a judicious use of the forceps should serve in the varied rounds of daily practice. The vestiges of the deciduous set, as relics of the infantile epoch, may need the forceps to aid where deranged or perverted nature has not normally completed its work. One or more stray temporary teeth will occasionally be found to persist in "holding the fort" long after their proper and typical leases have expired, the roots still remaining and obstinately opposing or deranging the status of the coming permanent teeth. Here, as a rule, extraction should come to the aid, and, as a dernier umpire, settle the question by ousting the tenant whose claims by limitation have been foreclosed. The tardy, deciduous tooth may give a mal-direction to the permanent that will be irremedial and compel the loss of the latter, if not worse consequences.

While I would severely rebuke the stupid and unprincipled slaughterer of teeth by wholesale extraction for the mere object of catering to the false fancy of the ignorant patient, or to satisfy the greed to make money upon, a low grade of practice and morals, I would wish to impress upon the minds of the deserving and honorable practitioner the true rationale of *not* sparing the forcep where it can so truly and effectually prevent or correct the irregularities or malformation, and especially in the treatment of the molars and bicuspids in youth—*never to devitalize a pulp of a first molar, or a tooth near by*, while the crowding for position of coming teeth still exists.—*Dental Luminary.*

